# Side Lights of the Erie Convention

**Published:** 1900-10

**Authors:** 


### SIDE LIGHTS OF THE ERIE CONVENTION.

President Harger will take a post-graduate course in the geography of Pennsylvania so as not to miss any more county secretaries.

Recording Secretary Marshall had some difficulty in finding a berth, owing to one of the gentler sex having pre-empted the one he had purchased.

Ex-President Jobson vouches for Congressman Sibley as a supporter of the bill for an army veterinary corps.

Chairman Irons proved a whole-souled entertainer, and was ably assisted by Drs. Jobson and Meredith.

Some seven new members were added at Erie to our already strong organization.

Our Association knows no political distinctions, and it gladly welcomed to membership Democratic Chairman Fry, of Centre County.

"Rattlesnake Joe," of Brookville, has a strong rival in the field of snakes in Corresponding Secretary Ranck.

Red-headed and hopeful ex-Secretary Rhoads was much missed, and his sonorous voice unheard along the shores of Lake Erie.

State Veterinarian Pearson has become quite a sketch artist, and loves to paint pictures of "Blazes Teddy Roosevelt" and pass around among the berths to awaken those whose slumbers linger after daylight.

Central Pennsylvania was well represented at Detroit and at Erie by Drs. Wier, Porter, Emery, Meredith, and William D. Wilson.

The sage of Reading, Dr. Noack, was one of the absent familiar faces at the semi-annual meeting at Erie.

Dr. Phillips, of North East, is a successful raiser of delicious grapes, and his great, big-hearted generosity would not let him forget the boys at Erie. He was lined up with Hoskins, of the JOURNAL, and the contrast in weights was well marked.

Dr. Bryce, of Erie, a graduate of Toronto, Class of 1870, could not withstand the temptation, and now will be a strong rival in Association stewardship.

Secretary Marshall, having become affluent and corpulent in veterinary practice, was several times mistaken for Mark Hanna.

Cleveland, Ohio, was represented by Dr. A. E. Cunningham; Dunkirk, N. Y., by Dr. C. H. Jewell; North Collis, N. Y., by Dr. John Gobin.

Drs. Pearson, Harger, Marshall, Ranck, and Hoskins represented Eastern Pennsylvania at Erie.

Where are the Rayners and Foelker? was many times asked.

City Veterinarian McNeil, of Pittsburg, was so completely overcome by the generous welcome at Detroit that he was unable to reach Erie to there tell his colleagues of the now famous meeting.

Coroner Porter, of Newcastle, never worries about inquests when Association meetings call him from home.

Member Irons, of Erie, was one of the strong advocates of the law passed in 1893 regulating the licensing of stallions.

